# Metabolic Compartmentalization at the Leading Edge of Metastatic Cancer Cells

**DOI:** 10.3389/fonc.2020.554272

**Published:** 2020-11-02

**Authors:** Kara Wolfe, Ryo Kamata, Kester Coutinho, Takanari Inoue, Atsuo T. Sasaki

**Affiliations:** ^1^ Division of Hematology and Oncology, Department of Internal Medicine, University of Cincinnati College of Medicine, Cincinnati, OH, United States; ^2^ Department of Cancer Biology, University of Cincinnati College of Medicine, OH, United States; ^3^ Division of Inflammation Research, Center for Molecular Medicine, Jichi Medical University, Tochigi, Japan; ^4^ Institute for Advanced Biosciences, Keio University, Tsuruoka, Japan; ^5^ Department of Cell Biology and Center for Cell Dynamics, Johns Hopkins University School of Medicine, Baltimore, MD, United States; ^6^ Department of Neurosurgery, Brain Tumor Center at UC Gardner Neuroscience Institute, Cincinnati, OH, United States

**Keywords:** membraneless metabolic compartmentalization, leading edge, liquid-liquid phase separation, metabolon, purine biosynthesis, GTP-metabolism, cancer, metastasis

## Abstract

Despite advances in targeted therapeutics and understanding in molecular mechanisms, metastasis remains a substantial obstacle for cancer treatment. Acquired genetic mutations and transcriptional changes can promote the spread of primary tumor cells to distant tissues. Additionally, recent studies have uncovered that metabolic reprogramming of cancer cells is tightly associated with cancer metastasis. However, whether intracellular metabolism is spatially and temporally regulated for cancer cell migration and invasion is understudied. In this review, we highlight the emergence of a concept, termed “membraneless metabolic compartmentalization,*”* as one of the critical mechanisms that determines the metastatic capacity of cancer cells. In particular, we focus on the compartmentalization of purine nucleotide metabolism (e.g., ATP and GTP) at the leading edge of migrating cancer cells through the uniquely phase-separated microdomains where dynamic exchange of nucleotide metabolic enzymes takes place. We will discuss how future insights may usher in a novel class of therapeutics specifically targeting the metabolic compartmentalization that drives tumor metastasis.

## Introduction

Many metastatic processes require dynamic changes in cell motility—i.e., epithelial-mesenchymal transition (EMT), detachment of cells from the primary tumor; local invasion of the basement membrane; intravasation and extravasation; or invasion in a distant site (1, 2). Genetic mutations and changes in transcriptional landscape that increase metastatic capacity have been identified [reviewed in (3, 4)]. EMT is one key initiating step for metastasis, converting epithelial cancer (i.e., carcinoma) to highly motile and invasive mesenchymal cell phenotype, rendering spatial asymmetry that corresponds to the emergence of lamellipodial and filopodial membrane extensions at the leading edge (i.e., the front end) (5–7), *via* dynamic signaling components. For example, localized activation of RAS and PI3K at the leading edge promotes cellular polarization, directional cell migration, and random cell migration (8, 9). Recent studies have demonstrated that rewiring of metabolic pathways in cancer and cancer stem cells *via* oncogenic signaling and/or EMT is another key regulator for cell motility and metastasis [reviewed in (10)]. However, the mechanism of this spatiotemporal regulation of metabolic enzymes in migrating cells remained unclear until recently (11). In this review, we highlight an emerging concept of membraneless metabolic compartmentalization and its possible roles in tumor invasion and metastasis.

## Membrane-Bound Organelles for Metabolic Compartmentalization

**Figure 1:**
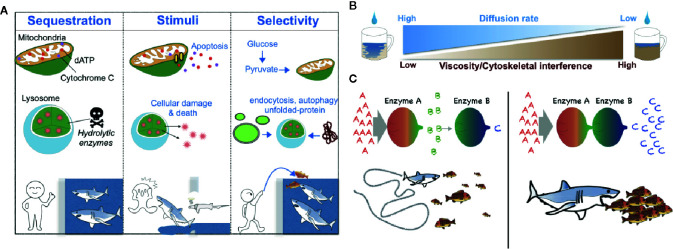
Schematic models of the biological roles of compartmentalization, viscosity, and local protein concentrations. **(A)** Metabolic compartmentalization by membrane-bound organelles confines metabolites to organelles to increase reaction efficiency and protect cellular contents—analogous to potential prey that may be protected from sharks by confining the predators to a shark tank (left). Release of organellar contents into the cytoplasm can elicit changes in cell fate (*e.g*., induction of an apoptotic program by cytochrome C and dATP or cellular damage mediated by lysosomal enzymes)—analogous to sharks that can either attack prey or themselves die when there is a breach in a shark tank (middle). Generally, the transportation of molecules into membrane-bound organelles is highly selective and regulated—analogous to sharks that may be fed without opening oneself up to the possibility of bodily harm by introducing prey into a shark tank from a distance (right). **(B)** A schematic diagram of the negative correlation between the fluid viscosity of a medium and the diffusion rate of metabolites within it. **(C)**. A schematic diagram of the effect of the local concentration or relative proximity of enzymes belonging to the same metabolic pathway. When Enzymes A and B are spatially separated, Enzyme B can receive only small amounts of its substrate b, which is generated by the distant Enzyme A. Thus, Enzyme B produces only small amounts of its enzymatic product c—analogous to a shark that can catch only a relatively small number of fish when the fish are sparse (left). However, when Enzymes A and B are in close proximity, Enzyme B can receive much more of its substrate b and thus produce much more of its product c—analogous to a shark that can capture more fish when the fish are schooling (right).

There are several critical roles for membrane-bound compartmentalization (12) (**Figure 1A**). For instance, metabolic compartmentalization within the peroxisome is crucial for sequestering toxic metabolites and for isolating the harsh conditions required for peroxisomal oxidative reactions from other more fragile cellular compartments (13). Sequestration of metabolic intermediates generally acts to preclude undesirable off-target enzymatic reactions and interference from other enzymatic pathways (14, 15) (**Figure 1A**); a network-based analysis conducted by Alam et al. suggests that the organelle-level compartmentalization of metabolic reactions relieves the inhibitory effect of unrestricted metabolite diffusion within cells by up to half (16). Additionally, changes in metabolic compartmentalization can evoke coordinated cellular responses such as apoptosis, in which case the release of cytochrome c from mitochondria into the cytosol initiates the apoptotic cascade (17) (**Figure 1A**).

## Membraneless Compartments for Metabolic Processes

Cell biologists of the late 19th and early 20th centuries considered the cytosol to be merely a “bag of enzymes” that functioned within the limits of diffusion and in which metabolites and proteins were free to randomly diffuse throughout the cell (18). However, accumulating evidence now points to the ability of cells to generate metabolic compartmentalization even in the cytoplasm. The cell may accomplish this form of compartmentalization through several mechanisms that derive from the physical nature of the cytoplasm, which differs locally in parameters of fluid viscosity, resulting in differential local diffusion rates of metabolites and proteins and differential local rates of molecular interactions (**Figure 1B**). In a term first coined by Paul Srere in 1985 (19), membraneless multi-enzyme complexes are referred to as *metabolons (* 20–22).

### Local Diffusion Rate

The cytoplasm of the cell is a viscous solution of ions, macromolecules, and cytoskeletal proteins. A number of metabolites could experience very slow diffusion rates in certain cellular contexts (18, 23–25). The diffusion rate of small molecules such as ions is reported to be reduced by less than two-fold in cytoplasm-like conditions compared to water (26), whereas the diffusion of larger macromolecules like nucleotides is hindered by greater than three-fold (27, 28). Likewise, the diffusion of polypeptides such as green fluorescent protein (GFP) has been found to be slowed by 3-14 times in bacterial cell cytosols compared to diffusion in water (29–31) and molecules larger than 60 kDa travel half the distance of smaller molecules (32). Additionally, increased nonspecific associations of larger macromolecules (e.g., proteins) with other solutes in the cytoplasm (e.g., polymerized actin, microtubules) are an impediment to diffusion (33–38) (**Figure 1B**).

The anomalous diffusion of macromolecules can be slowed by a tight mesh structure of actins and microtubules (**Figure 1**), which causes rapid jumps in the solute’s trajectory as it passes between contiguous actins and tubulin fibers (39). Furthermore, cross-linked actin filaments can transit into a gel phase, thereby dramatically increasing the elastic and viscous properties of the cytoplasm (40). Lastly, diffusion rates in the cytoplasm can also be impeded dramatically at spatiotemporal locations where liquid-liquid phase separation occurs (41).

### Local Protein Concentration

The increased local concentration of enzymes belonging to the same enzymatic pathway can protect highly labile metabolic intermediates *via* substrate channeling, which here acts as a mechanism to decrease reaction time (14, 22, 42) (**Figure 1C** **)**. To achieve substrate channeling, cells control the proximity of metabolic enzymes in an elegant way and thereby allow for tunable metabolic reactions that can respond dynamically to the evolving cellular status.

Several enzymes involved in glycolysis [e.g., glyceraldehyde 3-phosphate dehydrogenase (GAPDH), fructose-bisphosphate aldolase (ALDA), and phosphofructokinase (PFK)] have been discovered to be bound to actin fibers in the cellular cytoplasm, thereby forming long chains of metabolic enzymes belonging to the same glycolytic pathway that can promote rapid formation of pathway intermediates (20, 25, 42–47). In Hudder et al. the authors documented the large percentage of proteins that are bound to the polymerized filamentous actin (F-actin) cytoskeleton of CHO cells (20). Cell permeabilization released ~12% of the total protein content within the cell. By contrast, pre-incubating the cells with latrunculin B, which sequesters monomeric actin and thus diminishes the F-actin cytoskeleton, followed by permeabilization led to the release of nearly 40% of the proteins. This suggests that large fractions of proteins were bound to actin fibers (20).

## The Leading Edge as a Membraneless Organellar Compartment

The ruffling lamellipodium and actin-rich lamellum were described during the late 1960s in motile fibroblast cells in culture (48). As described in the previous section, the diffusion of nucleotides between the cytoplasm and the leading edge is likely hindered by the dense actin and microtubule mesh and the surrounding network of filament-bound macromolecules. Importantly, the formation and maintenance of a leading edge requires the input of purine nucleotides like guanosine 5’-triphosphate (GTP) and adenosine 5’-triphosphate (ATP).

The assembly of microtubules relies upon the binding of GTP to tubulin dimers, which are subsequently incorporated into the GTP-cap that resides at the plus-end of growing microtubules (49). Conversely, GTP hydrolysis results in GDP-bound tubulin dimers and its dissociation from the microtubules. Actin polymerization requires the binding of ATP to G-actin monomers, which may subsequently be incorporated into the barbed end of the growing F-actin polymeric chain. When ATP is hydrolyzed, the ADP-bound subunit conformation of the monomeric actin unit changes, leading to the dissociation of G-actin monomers from F-actin. In chick ciliary neuron culture, approximately 50% of global ATP hydrolysis is associated with the maintenance of the actin cytoskeleton (50).

The physical nature of the leading edge and the elaborated metabolic utility of purine nucleotides suggest a possible requirement for localized biosynthesis of ATP and GTP. Strikingly, the compartmentalization of an entire metabolic pathway within the leading edge was discovered in 2019 (11). In the following section, we will briefly introduce the GTP metabolic enzymes and their significance in cell motility.

## GTP Biosynthetic Enzymes in Cancer Cell Dissemination

### IMPDH

**Figure 2:**
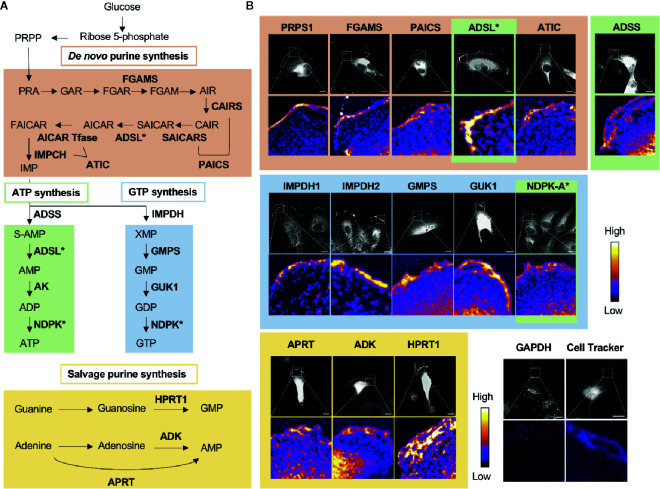
Nucleotide metabolic enzymes localize at the lamellipodial membrane. **(A)** Purine nucleotide biosynthesis schematic. The 9 enzymatic steps of *de novo* biosynthesis (orange), the ATP branch (green), GTP branch (blue), and selected salvage pathway enzymes (yellow). **(B)** Selected immunofluorescence staining images of purine biosynthetic enzymes (color scheme matches part A) localizing to the leading edge of migrating kidney cancer cells. Relative intensity map of immunofluorescence staining shown in bottom micrograph with intensity scale at the right. Immunofluorescence of GAPDH and fluorescence imaging of Cell Tracker dye, which stain intracellular proteins, show major signals in cytoplasm, which indicate that the localization of purine metabolic enzymes at the leading edge is specific. PRPS, phosphoribosyl pyrophosphate synthetase; FGAMS: phosphoribosyl formylglycinamidine synthase; PAICS, phosphoribosyl aminoimidazole succinocarboxamide synthetase; ADSL, adenylosuccinate lyase; ATIC, 5-amino-4-imidazolecarboxamide ribonucleotide transformylase/IMP cyclohydrolase; ADSS, adenylosuccinate synthase; AK, adenylate kinase; IMPDH, inosine-5′-monophosphate dehydrogenase; GMPS, GMP synthase; GUK1, guanylate kinase 1; NDPK, nucleoside-diphosphate kinase; APRT, adenine phosphoribosyltransferase; ADK, adenosine kinase; HPRT1, Hypoxanthine-guanine phosphoribosyltransferase; GAPDH, glyceraldehyde 3-phosphate dehydrogenase.

GTP and ATP can be synthesized through either the energy-saving salvage pathway or the *de novo* biosynthesis pathway (**Figure 2A**). The *de novo* biosynthetic process consumes glucose to synthesize the intermediate metabolite inosine 5’-monophosphate (IMP) after > 17 enzymatic steps. IMP is then used as a substrate by both the ATP-biosynthetic or GTP-biosynthetic branches of the *de novo* pathway. IMP dehydrogenase (IMPDH) is the rate-limiting, NAD^+^-dependent first step in GTP biosynthesis and commits IMP to the GTP pathway. IMPDH has been extensively studied in cancer biology since the 1950s (51–61), and is the *bona fide* target of the FDA-approved immunosuppressant mycophenolic acid (MPA) and its prodrug form mycophenolate mofetil (MMF, CellCept) (62), which is also used as an immunosuppressant in the clinic. There are two isotypes of IMPDH in humans, IMPDH isotype 1 (IMPDH1) and IMPDH isotype 2 (IMPDH2), of which IMPDH2 has been reported to be more highly upregulated in cancers (62–64).

Several studies show the functional role of IMPDH enzyme in cell motility. In human fibroblasts, inhibiting IMPDH *via* MPA treatment was shown to lead to decreased adhesion and migration along with dysregulated cytoskeletal proteins (65). Similarly, MPA treatment led to a decrease in the migration and invasion of gastric cancer cells *in vitro (* 66) and to a decrease in the EMT, *in vitro* migration, and metastatic seeding of prostate cancer cells (67). IMPDH inhibition was reported to decrease the fraction of GTP-bound RAC1, RHOA, and RHOC, the molecular switch proteins responsible for polarizing cells during migration (68), in melanoma cells (69). It is worth noting that many of these studies used long-term treatment with IMPDH inhibitor (e.g., over 16 h), which is likely to change both the transcriptional landscape and the phenotypic status (e.g., cell cycle arrest, apoptosis, or senescence) of the cellular targets. Nonetheless, although more careful analyses may be required to verify the direct effects of GTP on cancer cell motility, most of the evidence thus far points to a correlation between GTP biosynthesis and cell motility.

### Enzymes Downstream of IMPDH

The enzymes that lie downstream of IMPDH during GTP biosynthesis, GMP synthase (GMPS) and nucleoside diphosphate kinase-A (*NME1* or NDPK-A), a prominent member of the nucleoside diphosphate kinase family (70), have additionally been implicated in migration and metastasis. In human melanoma samples, increased GMPS protein expression was found in metastatic lesions compared to localized tumors, and the pharmacological inhibition of GMPS decreased melanoma cell proliferation and invasion, both *in vitro* and *in vivo (* 71). By contrast, NDPK-A expression in tumors has long been controversial. The gene name *NME1* [*NM23/NDP kinase* (non-metastatic clone 23)] (72) originated with the observed inverse correlation of its increased expression with decreased metastatic potential in mouse models and some human cancers (70, 73). However, the following studies showed that NDPK-A expression was correlated proportionally with metastasis in neuroblastomas (74) and renal cell carcinomas (75). Also, NDPK-A knockout in mice was observed to lead to diminished tumor formation *in vivo* and decreased lung colonization in xenograft models (76).

## Metabolic Compartmentalization of Purine Biosynthetic Enzymes Within the Leading Edge of Highly Motile Cancer Cells

In 2019, we found the striking localization of *de novo* purine biosynthetic enzymes as well as IMPDH1 and IMPDH2 at the membrane of the leading edge in metastatic renal cell carcinoma cell lines (11) (**Figure 2B**). All three enzymes of the GTP biosynthetic branch after IMPDH—GMPS, guanylate kinase (GUK1), and NDPK-A—were enriched significantly at the leading edge. Interestingly, we found a substantially greater colocalization at the leading edge of the cells in comparison to the cell body. Additionally, an essential enzyme for the GTP salvage pathway, HPRT1, was also enriched at the leading edge. Thus, all the GTP biosynthetic enzymes responsible for making GTP from IMP were localized at the leading edge (**Figure 2B** **)**.

While not all enzymes of the ATP biosynthetic pathway were tested, three of the four enzymes that act sequentially in the pathway to convert IMP to ATP were also found to enrich at the leading edge (**Figure 2B**). The ATP salvage enzymes APRT and ADK were also found to enrich at this location (11) (**Figure 2B**).

Given that all 16 enzymes analyzed localized to the leading edge and colocalized with IMPDH1 and/or IMPDH2, our data suggests the formation of a GTP- and possibly also an ATP-specific metabolic compartment—GTP and ATP metabolons, respectively—at the leading edge of the motile cancer cells. These compartments are expected to increase the local concentration of GTP and possibly also ATP *via* local, compartmentalized synthesis for availability to enhance actin polymerization, microtubule organization, and signaling. Consistent with this model, our recent cell biological and pathophysiological studies suggest a significant role for the non-membrane compartmentalization of purine metabolism at the leading edge in cell motility and the metastasis of certain types of cancers (manuscript *in preparation*).

## Translocation of a GTP Biosynthetic Enzyme to the Leading Edge Depends on F-Actin

Actin polymerization is required for the translocation of several proteins such as PI3K to the leading edge and the subsequent local induction of leading edge signaling (7, 77–80). Interestingly and mechanistically importantly, the enrichment of IMPDH1 and IMPDH2 at the leading edge was reduced following the inhibition of actin polymerization by latrunculin B treatment. The data suggest that F-actin polymerization could provide a mechanistic basis by which purine biosynthetic enzymes localize to the leading edge. Just as binding to actin filaments has been shown to enhance flux through the glycolytic pathway, it would be interesting to determine whether the enzymes involved in purine biosynthesis can directly bind to F-actin fibers during translocation and whether this potential binding leads to enhanced flux through the metabolic pathway. A potentially interesting experiment is to use inhibitors for F-actin or tubulin and assess the ATP and GTP distribution using their designated biosensors (81, 82). Future studies should clarify how F-actin polymerization induces IMPDH localization at the leading edge as well as whether the leading edge localization of other purine metabolic enzymes utilizes this actin-dependent localization.

## Heterogenous Purine Nucleotide Metabolism in Cell Membrane-Proximal Regions

ATP is one of the most abundant metabolites in a cell, ranging from 1 to 5 mM in mammalian cells (83). Intracellular distribution of ATP, detected by several types of genetic ATP biosensors (81), shows discrete ATP levels in organelles (84–87). There is also a report showing elevated ATP in the cortical region (88). Importantly, the turnover rate of ATP is increased at the leading edge *via* the activities of actin and tubulin polymerization (89), which is consistent with the previously noted high energy expenditure of cytoskeletal reorganization. Furthermore, very excitingly, the recently developed ratiometric fluorescent GTP biosensors have shown that the intracellular distribution of GTP is heterogeneous in SK-Mel-103 melanoma cells, with high GTP levels in the cytosol near certain regions of the cell membrane (82). Although further studies are required to eliminate potential confounding effects that might arise from the unintended pH sensitivity of these GTP biosensors, the provocative observation of high local GTP levels near some regions of the cell membrane, together with our identification of what is likely a GTP metabolon at the leading edge, suggest that elevated GTP levels specifically at the leading edge are highly probable.

## Possible Existence of Other Metabolons at the Leading Edge

Our results show that purine salvage enzymes, such as HPRT1, are also localized at the leading edge, raising the possible existence of metabolons of salvage ATP and GTP biosynthesis at the leading edge. In addition to the GTP and ATP metabolons, we expect that there are likely additional metabolons formed at the leading edge. For example, some key enzymes of glycolysis, 6-phosphofructo-2-kinase/fructose-2,6-biphosphatase 3 (PFKFB3), and pyruvate kinase isozyme M2 (PKM2), localize to the leading edge membrane in macrophages and to cellular projections in migrating tip endothelial cells (90). In the report containing this data, although the existence of no other glycolytic enzyme at the leading edge was confirmed, the authors proposed that the localization of such ATP-generating enzymes at the leading edge might promote concentrated ATP production to fuel the polymerization of actin, with which these enzymes heavily colocalize (90–92). Although our own data show that GAPDH, a key rate-limiting enzyme for glycolysis (93), fails to enrich at the leading edge formation in kidney cancer cells (11), it is possible that GAPDH may either translocate to the leading edge under certain conditions or that its accumulation at the leading edge may not be necessary given its high general abundance in the cell. Regardless, it remains important to further verify the existence of a glycolytic metabolon at the leading edge.

## Energy Homeostasis at the Leading Edge

Lastly, we highlight the possible links between homeostatic enzymes and leading edge activity. Adenylate kinase (AK)—which reversibly catalyzes the interconversion between 2ADP and 1ATP + 1AMP—is a critical enzyme for regulating cellular energy levels, and thus contributes to modulating the AMP-mediated response to stress signals (94, 95). In mouse embryonic fibroblasts, enforced localization of adenylate kinase 1 (AK1) to either focal contacts or the leading edge membrane significantly increases cell migration (96), supporting a notion of high metabolic turnover of ATP. Perhaps connecting to this observation, mitochondria have been shown to translocate to the leading edge through an AMPK-mediated mechanism to help sustain the local ATP:ADP ratio (97). The AMPK-dependent translocation of mitochondria to the leading edge suggests a locally enriched homeostatic system for ATP regeneration and recovery so that a high local ATP:ADP ratio may be maintained for robust leading edge activity. Currently, how AMPK induces the translocation of mitochondria remains largely unclear. Likewise, the dynamics of AMPK activation at the leading edge in relation to mitochondrial regulation remains unknown. Since AMPK is a known regulator of cell polarity (98), a possible model would be that downstream substrates of AMPK, which regulate cell polarity, may participate in the mitochondrial translocation. The use of AMPK biosensors may dissect the more detailed spatio and temporal regulation of AMPK activation and mitochondrial responses (99). Another potentially interesting experiment would be to compare the metabolic compartmentalization and ATP/GTP distribution in cells knocked out for AMPK or with mutations for the upstream kinase LKB1.

## Concluding Remark

The importance of the discoveries highlighted in this text and the significance of the idea that metabolic compartmentalization may crucially fuel the leading edge during cell migration is currently underappreciated. Additional studies are badly needed to determine whether pharmacological inhibition of the purine biosynthetic enzymes is sufficient to decrease cancer cell migration and invasion. For instance, it will be of prime importance to investigate whether metabolic compartments form within invadopodia—membrane protrusions on the cell surface of tumor cells that mediate matrix cleavage for tumor invasion—and, if so, their functional significance in tumor invasion. Also, it will be critical to clarify the interplay between localized purine metabolism at the leading edge and molecules essential for leading edge functions, such as membrane type-I-matrix metalloproteinase (MT1-MMP) (100), Na^+^/H^+^ exchanger NHE1 (101), and RHO GTPases, to name a few. Such interactions, if they exist, would further induce coordinated changes promoting cell migration and metabolic responses—e.g., normoxic HIF1 upregulation by MT1-MMP and MINT3 pathway (102–104). With ongoing advances in sub-cellular biosensor technology and targeted therapeutics, our appreciation of the biological importance and therapeutic potential of the leading edge purine metabolon is only just beginning.

## Author Contributions

KW and KC wrote the manuscript. RK projected to draw the illustration. TI overviewed. AS conceptualized, organized, and wrote the manuscript. All authors contributed to the article and approved the submitted version.

## Funding

The work is supported in part by Emerson Collective Cancer Research Fund (645098 to TI), MTP UC-Brain Tumor Center grant, Ohio Cancer Research grant, R21NS100077, and R01NS089815 (AS).

## Conflict of Interest

The authors declare that the research was conducted in the absence of any commercial or financial relationships that could be construed as a potential conflict of interest.
